# A healthy start: examining the contribution of caregiving quality to child physical health from birth to 14 years

**DOI:** 10.1007/s12144-025-08350-5

**Published:** 2025-09-06

**Authors:** Stefania V. Vacaru, Henrik Eckermann, Georgia Graat, Carolina de Weerth

**Affiliations:** 1 Donders Institute for Brain, Cognition and Behaviour, Department of Cognitive Neuroscience, Radboud University Medical Center, Kapittelweg 29, Nijmegen, 6525EN The Netherlands; 2 Department of Clinical Child and Family Studies & Amsterdam Public Health, Vrije Universiteit Amsterdam, Amsterdam, The Netherlands

**Keywords:** Infancy, Childhood, Adolescence physical health, Attachment security, Maternal sensitivity, Bayesian modelling, Low-risk

## Abstract

The attachment theory emphasizes the role of caregiver sensitivity in shaping children’s behavioral development. However, it remains unclear whether variations in early caregiving quality in low-risk populations influence children’s physical health across development. This study investigates whether maternal sensitivity and infant-mother attachment security are associated with children’s physical health from 0 to 14 years. We employed Bayesian generalized linear mixed models to analyze data from a low-risk Dutch sample from birth to age 14 (*N* = 193). Maternal sensitivity was assessed five times from 5 weeks to 14 years, while attachment security was assessed at 12 months using the Strange Situation Procedure. Child health complaints were recorded monthly in the first year and with intervals of 1–2 years until 14 years (*N* = 150) and categorized according to the International Classification of Primary Care: respiratory, skin, general, digestive symptoms. Higher maternal sensitivity scores in the first year of life and throughout 14 years were related to fewer total health complaints, particularly respiratory. Securely attached children showed fewer symptoms when early maternal sensitivity was higher. Insecurely attached children showed fewer health complaints, irrespective of sensitivity throughout childhood. Our findings indicate that maternal sensitivity and attachment may influence physical health. Future research should explore underlying psychobiological mechanisms.

**Supplementary Information:**

The online version contains supplementary material available at 10.1007/s12144-025-08350-5.

## Introduction

A parent’s availability and responsiveness to an infant’s needs are essential to survival (Ainsworth, 1964; Bowlby, 1979). Beyond the mere provision of care, the quality of caregiving, marked by parental sensitivity and emotional availability, profoundly impacts children’s lifelong psychophysical development (Doyle & Cicchetti, 2017; Raby & Dozier, 2015). Ground-breaking work on institutionalized children, exemplified by the Bucharest Early Intervention Project, documented that merely attending to an infant’s physical needswhile neglecting the emotional and cognitive needs, leads to severe, sometimes even irreversible psychological and physical damage (Fox et al., 2017; Humphreys et al., 2017). Conversely, sensitive and responsive caregiving equips infants with self-regulation capabilities, laying the foundation for a healthy life (Calkins & Leerkes, 2004; Pallini et al., 2018).

Sensitive caregiving reflects caregivers’ capacity to notice, understand, and appropriately and promptly react to their children’s cues (Ainsworth, 1969b) and is a core tenet of the attachment theory, which postulates that children of more sensitive mothers are more likely to form secure attachments (Atkinson et al., 2000; Bakermans-Kranenburg et al., 2003; De Wolff & Van Ijzendoorn, 1997). Continuous experiences with parental support help develop trust in the availability of parents, increasing the likelihood children will seek support during distress, rendering them more resilient (Bowlby, 1969). In secure attachment relationships, children feel safe and are confident in signaling distress and accepting help (Ainsworth, 1969b), contributing to physiological stress regulation. Nevertheless, the quest for understanding the contribution of early caregiving quality, namely parental sensitivity and infant-parent secure attachment to physical well-being throughout the lifespan, is underdeveloped, with most evidence testifying to the link between childhood attachment-related experiences and adverse psychological well-being (Thompson, 2000), as well as adverse psychoneuroimmunological outcomes (Asok et al., 2013; Bernard et al., 2019; Ehrlich, 2019; Ehrlich & Cassidy, 2021; Farrell et al., 2019). To bridge this gap, our prospective longitudinal study sought to examine the associations between caregiving quality, including maternal sensitivity and infant-parent attachment, and children’s common illnesses and health symptoms throughout infancy, childhood, and early adolescence.

###  Theoretical mechanisms: the attachment pathway

The mechanisms through which variations in the early caregiving environment may influence a child’s health are multifaceted and remain to be deciphered. One proposed mechanism suggests that caregivers exhibiting higher sensitivity are more attuned to their child’s well-being, enabling them to promptly detect initial symptoms, take appropriate actions, and expedite the infant’s recovery (Stern et al., 2020). Additionally, more sensitive caregivers can better attune to their children’s needs (Vacaru et al., 2022) and consequently help them regulate stress, leading to lower circulating cortisol concentrations (Loman & Gunnar, 2010). Higher childhood stress, particularly chronic, has been extensively shown to lead to altered patterns of endocrine discharge and often to a proinflammatory phenotype of the immune system, which over the life course may lead to hormonal dysregulations and possible adverse health outcomes (Dhabhar, 2014; Miller et al., 2011; Seiler et al., 2020). Supporting this pathway, insecurely attached children display higher cortisol concentrations than securely attached children when facing similar stressful events (Beijers et al., 2017; Groh & Narayan, 2019), providing evidence that early caregiving experiences shape physiological stress responses that may influence long-term health outcomes.

In sum, although the exact mechanisms through which early caregiving quality and parent-child attachment are related to physical health are yet to be determined, budding evidence indicates that the way children experience their early interactions with their primary caregiver(s) is associated with stress regulation and likely plays a role in shaping their physical health.

### Evidence from high-risk samples

The evidence to date mostly pertains to high-risk samples, defined as populations experiencing poverty, maltreatment, clinical interventions, significant trauma exposure, or institutionalization (Bernard et al., 2019; Fox et al., 2017). Contrarily, low-risk samples such as those of the current study are defined as populations without significant socioeconomic disadvantages, trauma exposure, or clinical health concerns. Findings to date in high-risk samples support the notion that poor early caregiving quality may result in altered biological processes during a crucial window of plasticity in development, which may, in turn, lead to poorer physical health. Indeed, insecure childhood attachment was related to higher indices of inflammation in a high-risk but not low-risk children sample (Bernard et al., 2019), and low early maternal sensitivity in a high-risk sample was linked to serious conditions, such as cardiometabolic risk, in adulthood (Farrell et al., 2019). Importantly, understanding the factors that influence mild health symptoms during childhood and adolescence may provide valuable insights into early health trajectories, even before they potentially progress into more severe medical conditions in adulthood. Moreover, the mechanisms linking caregiving quality to health outcomes may operate differently across these contexts, with more pronounced effects often observed in high-risk samples where multiple risk factors may interact. Gaps in our understanding are also due to reliance on cross-sectional studies or, in the case of longitudinal designs, reliance on convenience samples where studies do not always control for or consider crucial potential confounders (e.g., history of breastfeeding; Ehrlich, 2019; Ehrlich & Cassidy, 2021; Horta et al., 2023). Recently, there has been a plea for longitudinal evidence that includes relevant confounders and different assessments of early caregiving and markers of health starting in infancy (Ehrlich & Cassidy, 2021). In sum, there are many reasons why investigating links between early maternal caregiving quality and child health in well-controlled studies in low-risk samples is considered a valuable addition to the literature.

### Common childhood health symptoms

Common childhood symptoms, such as coughs, sore throats, and minor gastrointestinal disturbances, are typical experiences even for generally healthy children. Although non-life-threatening, examining these health issues may help uncover how early caregiving quality ‘gets under the skin’ in low-risk community samples, potentially affecting future physical and mental health. Waters and colleagues (2008) observed that the number of health complaints in children and adolescents was associated with poorer psychosocial well-being, social participation, and family functioning. Another extensive study (Houben-van Herten et al., 2015) involving 10,000 children found that (sub)optimal family environment (e.g., single-parent household, parental unemployment status) was a significant contributor to poorer children health and reduced life quality. However, this and another similar study (Ruijsbroek et al., 2011) conceptualized the family environment regarding socio-economic disparities. These disparities often relate to poorer nutrition, hygiene, and sleep quality (Poulain et al., 2020). Thus, it is essential to consider whether typical variations in caregiving quality contribute to children’s health independent of other adversities. To our knowledge, only one study testified to a significant positive contribution of early caregiving quality, operationalized through observations of maternal sensitivity as early as 5 weeks of the infant’s life, to fewer common health complaints that often do not require medical attention during the first year of life (Stern et al., 2020). These associations were found in a low-risk sample and above and beyond environmental contributors, such as maternal education, childcare attendance, number of siblings, breastfeeding, and maternal psychological well-being. These findings underscore the potential contribution of typical variation in caregiving quality to child health in low-risk contexts, which may be exacerbated in high-risk contexts. While Stern et al. (2020) examined the relationship between maternal sensitivity and infant health complaints exclusively during the first year of life, the current study on the same cohort substantially extends this work in several important ways. First, it expands the timeframe to cover a 14-year developmental period from infancy through early adolescence. Second, it incorporates multiple assessments of maternal sensitivity across development rather than focusing solely on early sensitivity. Third, it adds infant-mother attachment security as an additional dimension of caregiving quality. Finally, it employs more sophisticated statistical methods using Bayesian generalized linear mixed models to address the complexity of longitudinal health data. These extensions allow for examination of developmental trajectories and potential mechanisms that the previous study, with its narrower scope, could not address.

### Current study

This study investigated whether the quality of the caregiving environment in the first year and the history of caregiving throughout 14 years contributed to explaining variance in the physical health in this low-risk sample of children from birth to age 14. The analyses of Stern et al. (2020) for the first year were redone with a Bayesian Generalized Linear Mixed Models statistical method to make them comparable to the analyses for years 1–14. The comprehensive assessments of this sample allowed us to examine the contribution of (early) caregiving quality to physical health above and beyond other relevant caregiving practices (i.e., breastfeeding, center-based childcare) and family environment (i.e., maternal education, siblings). Accordingly, caregiving quality was indexed in two ways: (a) maternal sensitivity, both in the first year of life and throughout childhood and early adolescence, and (b) infant-mother attachment relationship, assessed at 12 months. We distinguished between caregiving quality in the first year of life (henceforth *early sensitivity*) and later assessment until age 14 (henceforth *sensitivity throughout childhood*), assuming that caring for an infant differs quantitatively and qualitatively from caring for an older child, posing different demands on the parents. These developmental differences may contribute differently to stress regulation and, thus, physical health, as an infant’s stress system is still immature and relies more on parental co-regulation. Physical health consisted of a broad range of common symptoms/illnesses, assessed from birth to 14 years.

Our first research question examined whether early caregiving quality, namely *(a)* maternal sensitivity in the first year of life and *(b)* attachment security, was associated with physical health. We proposed that *H1a*) greater maternal sensitivity in the first year of life would be associated with fewer health complaints throughout the first 14 years of life, and *H1b*) and higher infant-mother attachment security in the first year of life (continuous measure) would be associated with fewer health complaints throughout the first 14 years of life. Furthermore, given the paucity of research, we also explored whether different attachment classifications (secure/insecure or resistant, avoidant, disorganized) were related to variations in physical health.

Our second research question examined whether maternal sensitivity throughout childhood, namely from 0 to 14 years, was linked to children’s physical health. We hypothesized that *H2)* higher maternal sensitivity from 0 to 14 years would be linked to fewer health complaints in the same period. Finally, we also explored the interaction effect between maternal sensitivity and attachment security.

## Methods

###  Participants

This preregistered study (https://aspredicted.org/tt55z.pdf) belongs to the ongoing longitudinal BIBO study (Basal Influences on Child Development) investigating the psychobiological development of a predominantly white low-risk community sample of children from birth until 14 years of age. Data collection occurred in The Netherlands, where data on race/ethnicity cannot legally be collected if it is not part of the research question. Healthy pregnant women were recruited voluntarily through flyers handed out by midwife practices in The Netherlands. Prenatal inclusion criteria were an uncomplicated singleton pregnancy, no drug use during pregnancy, and no mental or physical health problems. Postnatal inclusion criteria were healthy full-term infants (*M =* 40.11 weeks, *SD =* 8.57 days) with a 5-min APGAR score ≥ 7 (*M =* 9.66, *SD =* 0.64). Out of 220 initial participants, eight were excluded due to birth complications, and 19 withdrew within the first three months due to personal reasons, resulting in a final initial dataset of 193 infant-mother pairs. Between 3 months and 14 years of age, 34 participants (18%) discontinued their participation for various reasons (e.g., moved countries). This led to 159 adolescent-mother dyads being invited for the 14-year measurement round, with 150 ultimately participating. Non-participation reasons included personal (*n* = 8) and COVID-related (*n* = 1). No significant differences in maternal age, maternal educational level, and child sex were found between the dyads who participated (*n* = 150), did not participate (*n* = 9), and dropouts (*n* = 34). Written informed consent was obtained from the mother, and ethical approval for the study was obtained from the Ethics Committee of the Faculty of Social Sciences (ECSW) of the Radboud University Nijmegen, the Netherlands (SW2017-1303-497, SW2017-1303-498, and ECSW-2018-067).

### Procedures

Around week 37 of pregnancy, mothers completed questionnaires on demographic information. Postnatally, throughout 14 years of the children’s lives, mothers reported on their children’s illnesses and health complaints through semi-structured interviews and questionnaires. During the first 12 months of life, data on infant illnesses and health complaints were obtained through monthly maternal interviews, including information on breastfeeding and center-based childcare attendance. At later ages, health-related information was obtained at 1–2-year intervals via questionnaires in which information was asked about the child’s frequency of symptoms and illnesses in the past year (see below for details). Information on the quality of caregiving was gathered for sensitivity via observations of (semi-structured) naturalistic age-appropriate interactions between mother and child and for attachment security via the Strange Situation Procedure. For sensitivity scoring, mothers and children were observed at 5 weeks, 12 months, 2,5 years, 10 years, and 14 years. At **5 weeks** of age (*M* = 33.5 days, *SD* = 4.9), mothers and their infants were filmed at home during a routine bath: the infant was undressed, bathed, and dressed again. At **12 months** (*M* = 53 weeks and 6 days, *SD* = 19 days), mothers and their infants visited the lab and were instructed to play together with four toys (i.e., hand puppets, books, and two types of puzzles) for 12 min in total (three minutes per toy). At age **2.5 years** (*M =* 30 months and 5 days, *SD* = 19 days), mothers and infants were filmed at home while playing with three toys (i.e., puzzles, blocks, and a fishing game) for 12 min in total (four minutes per toy). During a home visit at **10 years** (*M* = 10 years and 1 month, *SD* = 2 months), the mother and child were asked to discuss two different emotions for three minutes each and to play a Tangram game for six minutes. During a home visit at **14 years** (*M* = 14 years and 5 months, *SD* = 2 months), the mother and adolescent were asked to discuss two topics for three minutes each for the first interaction task. The topics were determined based on the 44-item issues checklist (Robin & Foster, 1989), which includes common discussion topics between parents and adolescents (e.g., low grades at school, how to spend money, and helping around the house). At the start of the home visit, the parent indicated per topic whether they had discussed it within the past four weeks ‘yes’ or ‘no.’ If they scored ‘yes,’ they rated on a 1–5 Likert scale how calm (1) or angry (5) the discussion about the topic made them feel. The two highest-scoring topics were used for the interaction task. For the second interaction task, adolescent-mother dyads were asked to organize and write down the details for an event of their choice for the child’s classmates (e.g., a sporting event, a party, an outing to a museum) for seven minutes.

### Instruments

***Maternal sensitivity*** at 5 weeks was coded using the 9-point Ainsworth scale (Ainsworth, 1969a), featuring the *Sensitivity* and *Cooperation* subscales. *Sensitivity* is the extent to which the mother responds to the infant’s needs and signals promptly and sensitively. Cooperation is how the mother adjusts her behavior to the infant and does not interfere with the infant’s ongoing activity. The scores were averaged as the two subscales were highly correlated (*r* = 0.86, *p* < 0.01), with higher scores representing higher caregiving quality. Trained students, unacquainted with the mothers and infants and blind to the data, rated the interactions, with 30% of the data double-scored to verify reliability (two-way mixed effects, relying on absolute agreement; Koo & Li, 2016). Substantial discrepancies (2-point difference on each subscale) between two observer scores prompted a third observer (i.e., an experienced senior observer) to score the video-recorded interaction independently. Interobserver reliability exceeded an intraclass correlation coefficient (*ICC*) of 0.90 for each construct.

Maternal sensitivity at 12 months, 2.5 years, 10 years, and 14 years was rated using the 7-point Erickson scale (Erickson et al., 1985). This scale replaced the earlier Ainsworth scale because it was deemed more appropriate for older ages. The scale allows for coding for maternal and child behaviours even at later ages, without the need for adaptations. Noteworthy, child behavior frequencies change, for instance “looking away” is more frequently observed in older ages, while “moving away” at younger ages. Interactions were rated for *Supportive presence*, defined as the extent to which the parent provides emotional support and confidence in the child, and *Respect of child autonomy*, defined as the extent to which the parent respects the validity of the child’s individuality, motives, and perspectives. Scores on the subscales featuring *Supportive presence* and *Respect for child autonomy* correlated significantly at 12 months (*r =* 0.62), 2.5 years (*r =* 0.46), ten years (*r =* 0.60), and 14 years (*r =* 0.75). We created a composite score of maternal sensitivity at each age, with higher scores representing higher caregiving quality. Videotapes were evaluated by at least two independent observers, with reliability assessed as described above for the 5-week observation. The intraclass coefficients were good for *Supportive presence* at 12 months (*ICC* = 0.95), 2.5 years (*ICC* = 0.91), 10 years (*ICC* = 0.97), and 14 years (*ICC* = 0.81) and for *Respect of child autonomy* at age 12 months (*ICC* = 0.70), 2.5 years (*ICC* = 0.70), 10 years (*ICC* = 0.93), and 14 years (*ICC* = 0.77).

The independent variables in this study were calculated as follows: *early maternal sensitivity* refers to mothers’ sensitivity to the infant in the first year of life and is the mean score of standardized scores of sensitivity at 5 weeks and 12 months assessments; *maternal sensitivity throughout childhood* refers to the history of the mother’s sensitivity from 0 to 14 years and is the mean score of standardized scores of sensitivity at 5 weeks, 12 months, 2.5 years, 10 years, and 14 years assessment rounds. Higher scores denote higher caregiving quality. The rationale for computing *early maternal sensitivity* separately to reflect the first year of the infant’s life is that this first year had a much higher assessment frequency of health symptoms than the following years, and that this led to performing separate analyses (see details below).

***The infant-mother attachment*** was assessed using the Strange Situation Procedure (Ainsworth, 1969b) during a laboratory visit at 12 months of infant age. Infants were observed and video-recorded during the 20-minute procedure, which consisted of exposing the infants to an unfamiliar environment, the appearance of a stranger in the room, and two separations from the mother. These consecutive mildly stressful situations are supposed to be increasingly disturbing for the infant (Ainsworth & Bell, 1981). Tapes of all the procedures were coded at the Institute of Child Development, University of Minnesota, USA. The coders were trained and supervised by expert Dr. Elizabeth Carlson. A subset of 46 infants’ tapes was double-coded, reaching good interobserver reliability (Cohen’s *K* = 0.82, *ICC* = 0.86), based on the 4-way classification data (secure, avoidant, resistant, disorganized). A small number of these ratings (*n* = 5; 0.65%) were missing and imputed using the expectation-maximization algorithm (Bolhuis et al., 2022; Dempster et al., 1977). Subsequently, ratings of infant behaviors during the two reunions (proximity, contact maintaining, resistance, avoidance) were used to compute a continuous score for attachment insecurity, following Van Ijzendoorn and Kroonenberg’s algorithm (Van Ijzendoordn & Kroonenberg, (Ijzendoorn, et al., 1990)). The algorithm entails weighing each reunion episode and the mean scores of each behavior in contributing to the final security score. Thus, higher continuous scores reflect greater attachment insecurity (Bolhuis et al., 2022). Alongside, we employed the commonly used dichotomous operationalization of attachment insecurity which classifies infants’ 4-way classification into secure or insecure (avoidant, resistant, or disorganized). Secure attachment was coded as 0 (henceforth securely attached children) and insecure as 1 (henceforth insecurely attached children).

#### Physical health

Because in the local Dutch cultural context, doctor visits for minor health symptoms are infrequent, parent reports offer a more valid representation of common child health symptoms than medical records. Hence, infant physical health was assessed monthly in the first year by trained researchers conducting semi-structured interviews with mothers: three face-to-face interviews at one, five, and 12 months, and the other nine via telephone. Mothers reported the health symptoms of their infants during the previous month, using a 24-item checklist to enhance their recall and scoring validity. After an initial open-ended question (“Has your child been sick during the past month?”), mothers were asked to respond with “yes” or “no” to the presence of 24 common infant illnesses and health complaints (Table 1). Additionally, mothers quantified the frequency of each symptom experienced by their infant over the past month. To reduce the amount of data in the first year, complaints were averaged over the trimesters in the first 12-month period, resulting in four health assessments in the first year (Beijers et al., 2011). These four health assessments were then summed into one score denoting physical health in the first year. If one month in a trimester was missing, the mean trimester score was calculated by averaging the remaining two months. If two of the three months were missing, the child’s trimester score was considered missing, and the child was taken out of the analyses (*n* = 5). Health data were categorized based on the International Classification of Primary Care (ICPC; Lamberts & Wood, 1987; Wood et al., 1992) into four categories: respiratory, skin, digestive, and general symptoms. In this study, we also created a total symptom score by summing up all the subcategories’ counts for each year. The ICPC classifies illnesses and complaints into non-overlapping domains and is a widely recognized method in infancy and childhood research (Soler et al., 2008; Zijlmans et al., 2017).

**Table 1:** Illnesses and health complaints, according to the international classification of primary care, are divided by category

Respiratory	Digestive	General	Skin
**R05** Cough	**D01** Abdominal pain/cramps, general	**A03 Fever** ^**3**^	**S09** Infected finger/toe
**R07 Sneezing/nasal congestion** ^**1**^	**D03** Heartburn	**N01** Headache	**S74** Fungal infection
**R25** Mucus	**D10 Vomiting** ^**3**^	**A04** Tiredness	**S29** Skin symptom/complaint
**R02** Shortness of breath/dyspnea	**D11 Diarrhea** ^**4**^	**A74 Rubella** ^**5**^	**S74 Dermatophytosis** ^**3**^
**R04** Breathing issues	**D12 Constipation** ^**5**^	**A72 Chickenpox** ^**7**^	**S01** Cold sore
**R74** Cold	**D13 Jaundice** ^**2**^	**A71** Measles	**S84 Impetigo** ^**3**^
**R71 Whooping cough** ^**2**^	**D71** Mumps	**A76 Viral exanthema**,** other**^**3**^	**S86 Dermatitis seborrhea** ^**3**^
**R72 Strep throat** ^**3**^	**T99** Lactose intolerance	**A76** Erythema infectiosum	**S87 Dermatitis/atopic eczema** ^**3**^
**R74 Upper respiratory infection acute** ^**3**^	**D16** Rectal bleeding	**A76** Roseola infantum	**S88 Dermatitis contact/allergic** ^**3**^
**R77** Laryngitis/tracheitis acute	**D20** Mouth/tongue/lip symptom/complaint	**H01** Ear pain/earache	**S89** Diaper rash
**R78 Acute bronchitis/bronchiolitis** ^**3**^	**D22** Worms/other parasites	**H04** Ear discharge	**S85** Pilonidal cyst/fistula
**R80** Influenza	**D73** Gastroenteritis presumed infection	**H29** Ear symptom/complaint	**S22** Nail symptom/complaint
**R81 Pneumonia** ^**3**^	**D99 Disease digestive system**,** other**^**3**^	**F01** Eye pain/eye ache	**S10** Boil/carbuncle
**R96 Asthma** ^**3**^		**F03** Eye discharge	**S02** Pruritus
**R97 Allergic rhinitis** ^**4**^		**F29** Eye symptom/complaint	**S06** Rash localized
**R25** Sputum/phlegm abnormal		**F73** Eye infection/inflammation, other	**S01** Pain/tenderness of skin
**R93** Pleura fluid, other		**H71 Otitis media acute/myringitis** ^**3**^	
		**A77** Viral disease, other	
		**A78** Infectious disease, other	
		**A94** Perinatal morbidity, other	
		**F80** Blocked lacrimal duct of infant	
		**A12 Allergy to wheat** ^**4**^	
		**A12 Allergy to house dust** ^**4**^	
		**A12 Allergy to dust mites** ^**4**^	
		**A12 Allergy to medication** ^**4**^	
		**A12 Allergy to pets and animals** ^**4**^	
		**A12 Allergy to mold and fungus** ^**4**^	

To accommodate the low incidence of ear- and eye-related issues, these were grouped with general illnesses and complaints. The superscript numbers indicate at which age symptoms were asked. No superscripted symptoms indicate that these symptoms were asked at each age. ^1^All ages between 0–6 years; ^2^All ages, except year 10 and 11; ^3^All ages between 7–14 years; ^4^All ages, except year 11; ^5^All ages, except year 8; ^6^All ages, except year 10, 11, and 14; ^7^All ages, except year 11 and 14. The 24 symptoms we used to assess health in our checklist are highlighted in bold.

The frequency of infant illnesses and health complaints in the following measurement rounds was assessed (with one exception) by questionnaires with open questions and checkboxes for symptoms/illnesses in line with the interviews of the first 12 months. This was done with a pencil and paper questionnaire at ages 2.5 (*n* = 178), 4 (*n* = 179), and 5 (*n* = 176). At the age of 6 (*n* = 160), the assessment was done during a phone interview, whereas at ages 7 (*n* = 171), 8 (*n* = 172), 10 (*n* = 149), 11 (*n* = 136), 12.5 (*n* = 150), and 14 (*n* = 151) years, the information was gathered via online questionnaires. At each measurement round, the mother reported on the child’s health over the past 12 months. Some questions were adapted to reflect the most common symptoms/illnesses for a specific age: scarlet fever and 5th and 6th diseases were left out from age 10 onwards (but note that these could be reported in the general question about ‘other’ health issues to report).

Answers to the questions from birth to 14 years were coded in a standardized manner, with 0 indicating never experienced a symptom and 12 indicating monthly recurrence or that the symptoms never resolved over the preceding 12 months. Due to varying assessment frequencies, with monthly assessments in the first year and in intervals of 1–2 years after, the symptom counts in the first year exceed those of subsequent years. The health data were examined by three independent raters, with excellent interclass correlation coefficients (ICCs) ranging from 0.99 to 1.

### Covariates

The covariates considered in this study were chosen to keep the current investigation comparable to the previous studies investigating similar relations at different ages (Stern et al., 2020; Zijlmans et al., 2017). These encompassed infant sex (assigned at birth), maternal education level, breastfeeding duration in the first year, number of siblings, and center-based childcare attendance in the first 12 months of life. Infant sex was coded as 0 for girls and 1 for boys. Maternal education level, a proxy for socioeconomic status (SES), was determined at 37 weeks of pregnancy. Education levels were categorized 1 for ‘low-medium’ (primary school to secondary and pre-university education) and 2 for ‘high’ (higher vocational education, university, and higher). Breastfeeding duration in the first year was recorded using a weekly logbook during the first 6 months of the infant’s age, supplemented by a follow-up interview at 12 months. The total weeks of breastfeeding were converted into months for analysis. The number of siblings at the time of the infant’s birth was categorized as 0 for ‘no siblings’ and 1 for ‘one or more siblings.’ Center-based childcare attendance was assessed through monthly maternal interviews during the first year of life and represented as 0 for ‘no’ and 1 for ‘yes’ (childcare attendance in the first year).

### Statistical analysis

All analyses were performed in the *R* programming language (R Core Team, 2022), and the code is publicly available ( https://doi.org/10.5281/zenodo.10401249). The following variables had missing data: childcare (1.55%), education level (4.14%), siblings (2.07%), breastfeeding duration (3.11%), attachment security (4.15%), maternal sensitivity at 5 weeks (1.1%), at 12 months (5.18%), at 2.5 years (3.11%), at 10 years (19.68%), at 14 years (28.49%), health symptoms (< 15% for each of the assessment moments). We used multiple imputation (predictive mean matching; m = 50) using the *mice* package (Van Buuren & Groothuis-Oudshoorn, 2011) before model fitting. Predictive mean matching remains robust even when the imputation model is misspecified and tends to perform as well as, or better than, standard parametric imputation methods across different missingness scenarios, particularly with up to 20–30% missing data (Kleinke, 2017; Marshall et al., 2010). The brms package allows multiple imputed data sets as input. The posterior distribution is then based on all imputed data sets, reflecting the uncertainty and additional variation implied by the imputation model. We performed sensitivity analyses using complete case analysis and reported if the results differed meaningfully. As preregistered, we investigated whether the assumptions underlying a hierarchical linear regression model held. Since several assumptions were violated across measurements, we decided to fit a Bayesian Generalized Linear Mixed Model (GLMM) with the Poisson family using the *brms* package (Bürkner, 2017). We built the models using a stepwise procedure as follows. The first step involved prior predictive simulations through a repetitive process in which models with different prior distributions were created (not using the data) and then used to generate artificial symptom counts. Proper selection of prior distributions is necessary for optimal model performance and convergence in complex multilevel models (McElreath, 2018). The goal was to identify priors that are not strongly regularizing but avoid unrealistic relationships or outcomes. For example, flat priors would have resulted in artificial symptom counts > 1e^15 in prior predictive checks. Note that none of the resulting priors was strongly regularizing, and we performed sensitivity analyses to investigate results using flat priors. Results did not differ regarding rejections of null hypotheses. However, models with flat priors would encounter more problems during model fitting (divergent transitions) and would, therefore, have been less reliable, as expected. The second step involved building a properly working base model before including the variables to test our hypotheses. We increased model complexity step-by-step using posterior predictive checks and leave-one-out cross-validation (LOO) (Vehtari et al., 2017) as guidance (variables were only included if they improved model fit based on LOO while posterior predictive checks pointed out model weaknesses). The model-building process is documented in the R code file *poisson_models.R.*

The resulting model fit the data very well according to posterior predictive checks and can be described as:

$$\begin{aligned} \begin{array}{c}\:symptoms_i\sim\:Poisson\left(\lambda\:\right)\\\:log\left({\lambda\:}_i\right)={\alpha\:}_{id_i}+{\beta\:}_1year_i+{\beta\:}_2year_i^2\\+{\beta\:}_3firstyear_i+{\beta\:}_4firstyear_i\times\:childcare_i\\+{\beta\:}_5firstyear_i\times\:breastfeeding_i+{\beta\:}_6edu_i+{\beta\:}_7siblings_i\\+{\beta\:}_8sensitivity_{year_j}\\\:{\alpha\:}_j\sim\:N\left(\overline\alpha,\sigma\:\right)\\\:\overline\alpha\sim\:N\left(2,0.75\right)\\\:\sigma\:\sim\:Exp\left(1\right)\\\begin{array}{c}\:{\beta\:}_1\sim\:N\left(-0.15,0.15\right)\\\:{\beta\:}_{2,3,4...8}\sim\:N\left(0,0.5\right)\\\:{\beta\:}_k\sim\:N\left({\overline\beta}_8,\gamma\:\right)\\\:\gamma\:\sim\:Exp\left(1\right)\end{array}\end{array} \end{aligned}$$

where $\:{\beta\:}_{8}$ corresponds either to one of the maternal sensitivity (early maternal sensitivity and maternal sensitivity throughout childhood) variables or to one of the three attachment variables (continuous score, dichotomous score, categorical scores). For $\:{\beta\:}_{8}$, we allowed the slope to vary between each year (random slopes). A model with varying slopes fitted the data better most of the time, while a model with varying intercepts between individuals (random intercepts) provided better model fit all the time. Note that the most complex model (described above) was sometimes outperformed by a simpler model that omitted siblings, education, breastfeeding, or the random slopes, depending on which of the sensitivity/attachment variables or outcomes was used. While the conclusions do not differ regarding rejections of null hypotheses, we report the parameter estimates derived from the best-fitting model here. Posterior distributions of all imputed datasets were combined before evaluating the hypotheses. As preregistered, we utilized the *hypothesis* function of the *brms* package as a decision criterion. The reported p-value is the posterior probability as output by the *hypothesis* function and, in this specific case, represents the probability of the effect being larger or smaller than zero, depending on the direction of the hypothesis. We rejected the null hypothesis if this probability exceeded 0.95. We adhered to the convention of reporting *β* coefficients and statistics that indicate whether the null hypothesis could be rejected. However, deriving meaning from *β* coefficients from GLMMs is challenging compared to linear regression models. Therefore, to provide a more intuitive description of the magnitude of the association, we provide a graph for each model using the total symptom counts for each year. The graph shows the median posterior prediction per year (to illustrate how the association varies between the years) and per level (−2SD, −1SD, 0, +1SD, +2SD) of the variable for which we try to interpret the association. To calculate the median posterior prediction per year and level of the variable, we generated predictions for varying levels of all the covariates with breastfeeding set to the median. In other words, the graphs show what the model predicts when we vary our variable of interest (across various combinations of the covariates). Note that while the median is suitable to summarize the posterior predictive distribution to a point estimate, we do not show prediction intervals in the graph to avoid overplotting.

## Results

### Preliminary analyses

Sample characteristics and descriptive statistics of the predictors are summarized in Table 2. Most mothers were highly educated (74.1%), married/cohabitating (97.9%) and multiparous (57%). Infant sex distribution was balanced in our sample (52% boys). On average, breastfeeding duration in the first year lasted approximately 5 months, and nearly 60% of infants attended center-based childcare during their first year. Maternal mean sensitivity scores were consistently moderate to high across all assessed ages. At 12 months,4% of the infants did not contribute data on attachment; 65.4% of infants were classified as securely attached, while 10.9% were categorized as resistant, 5.2% as avoidant, and 14.5% as disorganized in the insecurely attached group.

**Table 2:** Descriptive characteristics of mothers and their children

	*N*	%*	Mean	SD	Range
Maternal age at delivery *(years)*	193		32.51	3.64	21.1–42.9
Maternal education level					
Low-medium	42	21.8			
High	143	74.1			
Marital status (married/cohabitating)	193	97.9			
Infant sex					
Boys	101	52.3			
Girls	92	47.7			
Number of siblings					
0	79	40.9			
≥ 1	110	57			
Breastfeeding duration 1 st year *(months)*	187		5.43	4.31	0.00–12.00
Center-based childcare attendance (yes)	190	59.6			
Maternal sensitivity^1^					
5 weeks	191		5.48	2.07	1.00–9.00
12 months	183		4.55	1.04	1.60–7.00
2.5 years	187		5.49	0.67	3.60–6.80
10 years	155		5.57	0.77	3.00–7.00
14 years	138		5.12	1.18	1.00–7.00
Attachment security					
Continuous score	185		0.56	0.65	−5.45–5.32
Secure	126	65.4			
Insecure	59	30.6			
Avoidant	10	5.2			
Resistant	21	10.9			
Disorganized	28	14.5			

*SD* standard deviation. *Not all variables add up to 100% due to missingness. ^1^All sensitivity scores range between 1–7, except for 5 weeks assessment that ranges from 1–9.

Correlation analyses (Table 3) between demographic information and predictors indicate that higher maternal education is associated with higher maternal sensitivity at all ages, except at 12 months of infant age. Additionally, higher maternal sensitivity at 12 months of age correlated positively with a longer duration of breastfeeding. Further, attachment security at 12 months did not correlate with maternal sensitivity at any point. Maternal sensitivity assessed at different ages was not or only weakly correlated over adjacent ages.

**Table 3:** Correlation analyses of demographic information of the sample and predictors in the study

	1.	2.	3.	4.	5.	6.	7.	8.	9.	10.	11.	12.		
1. Center-based childcare attendance	**-**													
2. Child sex	− 0.003	-												
3. Maternal education	0.021	**− 0.149** ^*^	-											
4. Number of siblings	− 0.045	0.039	− 0.088	-										
5. Breastfeeding duration 1 st year	0.104	0.040	0.101	0.045	-									
6. Attachment dichotomous	0.026	− 0.021	− 0.010	0.147*	0.017	-								
7. Attachment security (continuous)	0.069	− 0.060	0.044	− 0.022	0.077	**− 0.562** ^**^	-							
8. Maternal sensitivity 5 weeks	− 0.072	− 0.038	**0.173** ^*^	− 0.090	− 0.013	− 0.080	0.119							
9. Maternal sensitivity 12 months	0.022	− 0.055	− 0.005	0.022	**0.182** ^*^	0.004	0.052	**0.167** ^*^	-					
10. Maternal sensitivity 2.5 years	0.006	− 0.105	**0.158** ^*^	− 0.028	0.032	− 0.062	0.016	0.007	0.137	-				
11. Maternal sensitivity 10 years	− 0.024	0.008	**0.174** ^*^	0.020	− 0.027	0.075	− 0.091	0.023	0.089	**0.165** ^*^	-			
12. Maternal sensitivity 14 years	0.079	0.133	.174^*^	− 0.121	0.077	− 0.019	− 0.005	0.118	0.024	0.071	**0.219** ^*^	-		

Childcare attendance is coded as 0 = no attendance, 1 = yes attendance. Sex is coded as 0 = boys, 1 = girls. Maternal education is coded as 1 = medium-low and 2 = high education. The attachment dichotomous variable is coded as 0 = secure, 1 = insecure; a higher continuous attachment score refers to higher attachment security. The significance level was set at < 0.05. * *p* < 0.05, **p < 0.01. Significant correlations are highlighted in bold. The grey shading highlights the associations between caregiving variables.

For illustration purposes, we plotted the z-scores of maternal sensitivity between secure and insecure children throughout time (Supplementary Fig. 1). Furthermore, we reported maternal sensitivity’s raw means and standard deviations for all four attachment categories (Supplementary Table 1). The repeated measures ANOVA test showed no significant differences in maternal sensitivity as a function of attachment category or time (*p* > 0.104).

**Fig. 1:**
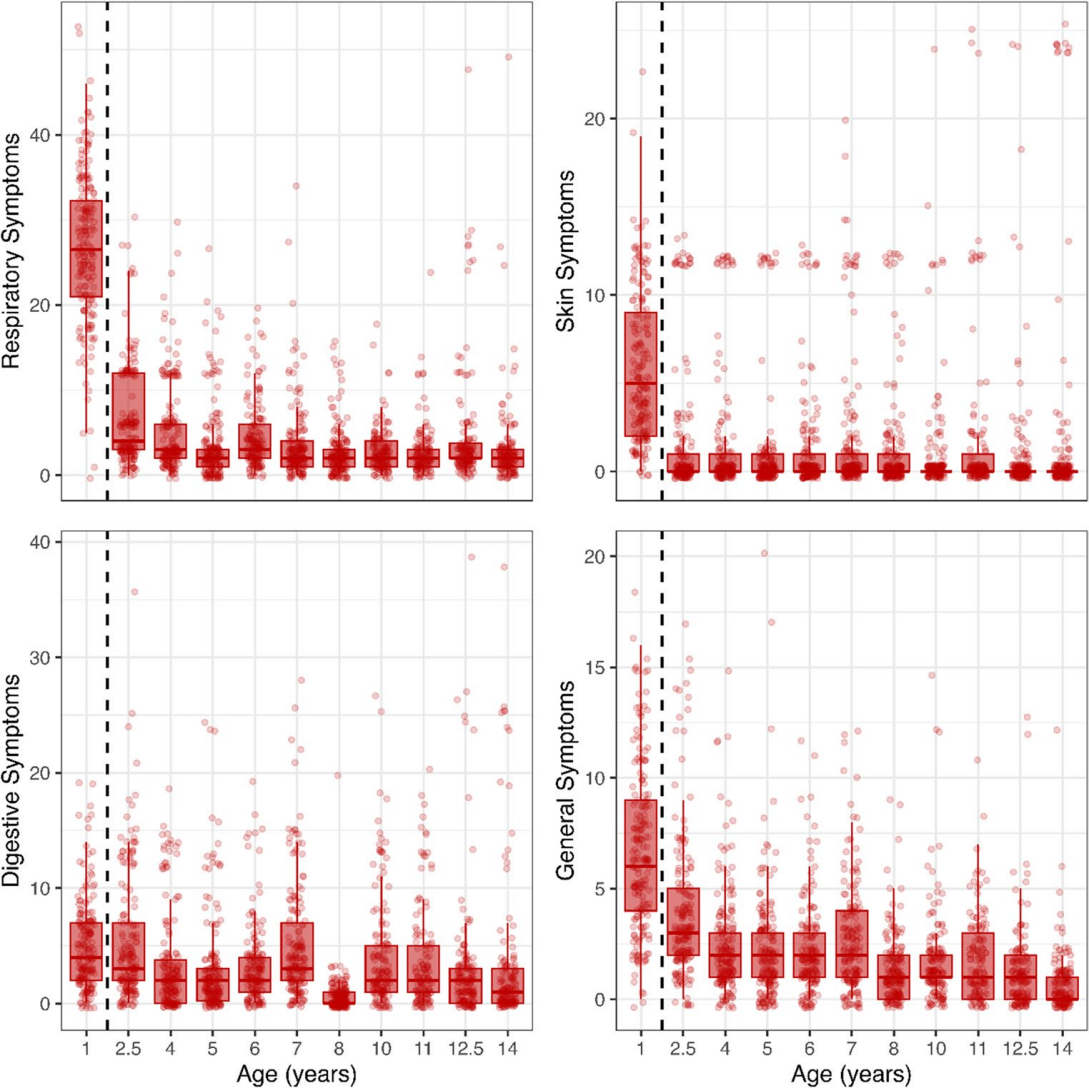
Raw data points across age for each symptom category. The y-axis represents the number of symptoms, and the x-axis is the age in years. Numbers in the first year are not comparable to those of the remaining years because of the different data collection frequencies (see Methods)

The mean of the total health score and subcategories (Supplementary Table 2) is highest in the first year because these scores represent the sum of symptoms/illnesses over 12 months, as explained earlier. The total health score remains relatively consistent across all measurement rounds after the first year. Figure 1 illustrates that while respiratory and general symptoms tend to decrease over time, the same trend does not apply to the digestive and skin categories.

### Main analyses

#### Early maternal sensitivity

**Fig. 2:**
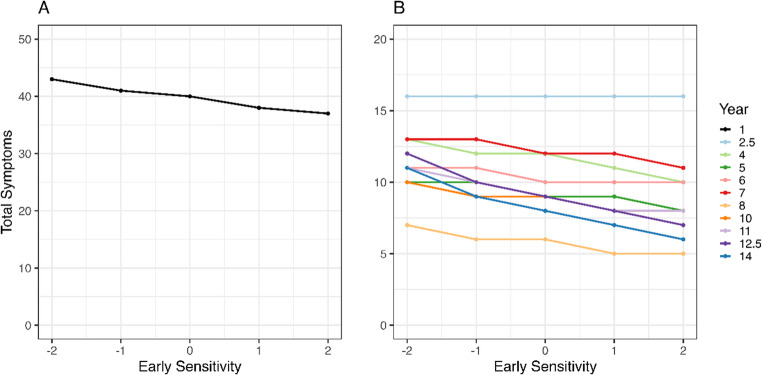
Median posterior predictions illustrate the effect size for the relation between early maternal sensitivity (x-axis) and total symptom counts (y-axis) for each year. The first year (**A**) is shown separately from the other years (**B**)

While our data hinted that more sensitive mothers in the first year of their infant’s life were more likely to report fewer total health symptoms for the children compared to less sensitive mothers, we could not reject the null hypothesis ($\:\beta\:$=−0.043, 95% CI=[−0.769; 0.008], *p* = 0.949). The analysis of the separate symptoms revealed that this result was mainly due to lower respiratory symptoms ($\:\beta\:$=−0.068, 95% CI=[−0.203; −0.009], *p* = 0.981), for which we rejected the null hypothesis. While other symptoms also decreased on average with increasing maternal sensitivity in the first year, the null hypothesis could not be rejected for skin ($\:\beta\:$=−0.061, 95% CI=[−0.454; 0.079], *p* = 0.785), digestive ($\:\beta\:$=−0.036, 95% CI=[−0.348; 0.073], *p* = 0.701) or general symptoms ($\:\beta\:$=−0.008, 95% CI=[−0.154; 0.057], *p* = 0.608). Figure 2 shows that for total health symptoms, depending on the year, mothers who are less sensitive (−2SD) are expected to report 6 (Fig. 2A) and between 2 and 5 (Fig. 2B) more symptoms compared to more sensitive mothers (+ 2SD) in the first and subsequent years, respectively. Results are summarized in Supplementary Table 3.

#### Maternal sensitivity throughout childhood

**Fig. 3:**
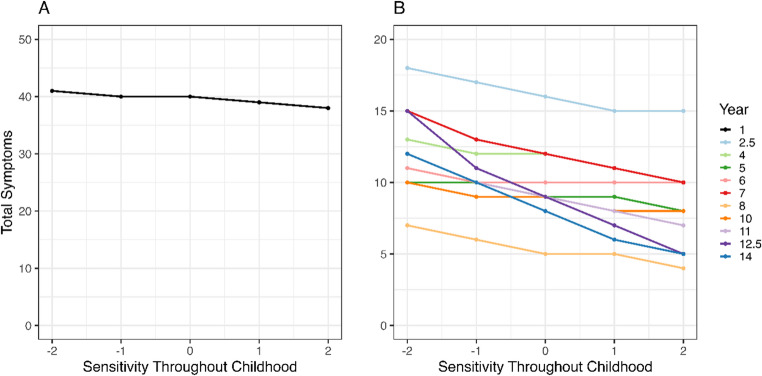
Median posterior predictions illustrate the effect size for the relation between maternal sensitivity throughout childhood (x-axis) and total symptom counts (y-axis) for each year. The first year (**A**) is shown separately from the other years (**B**)

We observed similar results when using the average scores of maternal sensitivity throughout childhood, with fewer expected total health symptoms ($\:\beta\:$=−0.095, 95% CI=[−0.440; −0.022], *p* = 0.988) and respiratory symptoms ($\:\beta\:$=−0.110, 95% CI=[−0.348; −0.040], *p* = 0.994) with increasing maternal sensitivity. However, here we also found that maternal sensitivity throughout childhood was negatively related to digestive symptoms ($\:\beta\:$=−0.142, 95% CI=[−0.475; −0.015], *p* = 0.955). The null hypotheses could not be rejected for skin ($\:\beta\:$=−0.005, 95% CI=[−0.560; 0.179], *p* = 0.481) and general symptoms ($\:\beta\:$=−0.025, 95% CI=[−0.333; 0.063], *p* = 0.660). Figure 3 presents the results for total health symptoms. It illustrates that less sensitive mothers (−2SD) are expected to report 3 (Fig. 3A) and between 1 and 10 (Fig. 3B) total symptoms more than more sensitive mothers (+ 2SD) in the first and subsequent years, respectively.

#### Infant-mother attachment

**Fig. 4:**
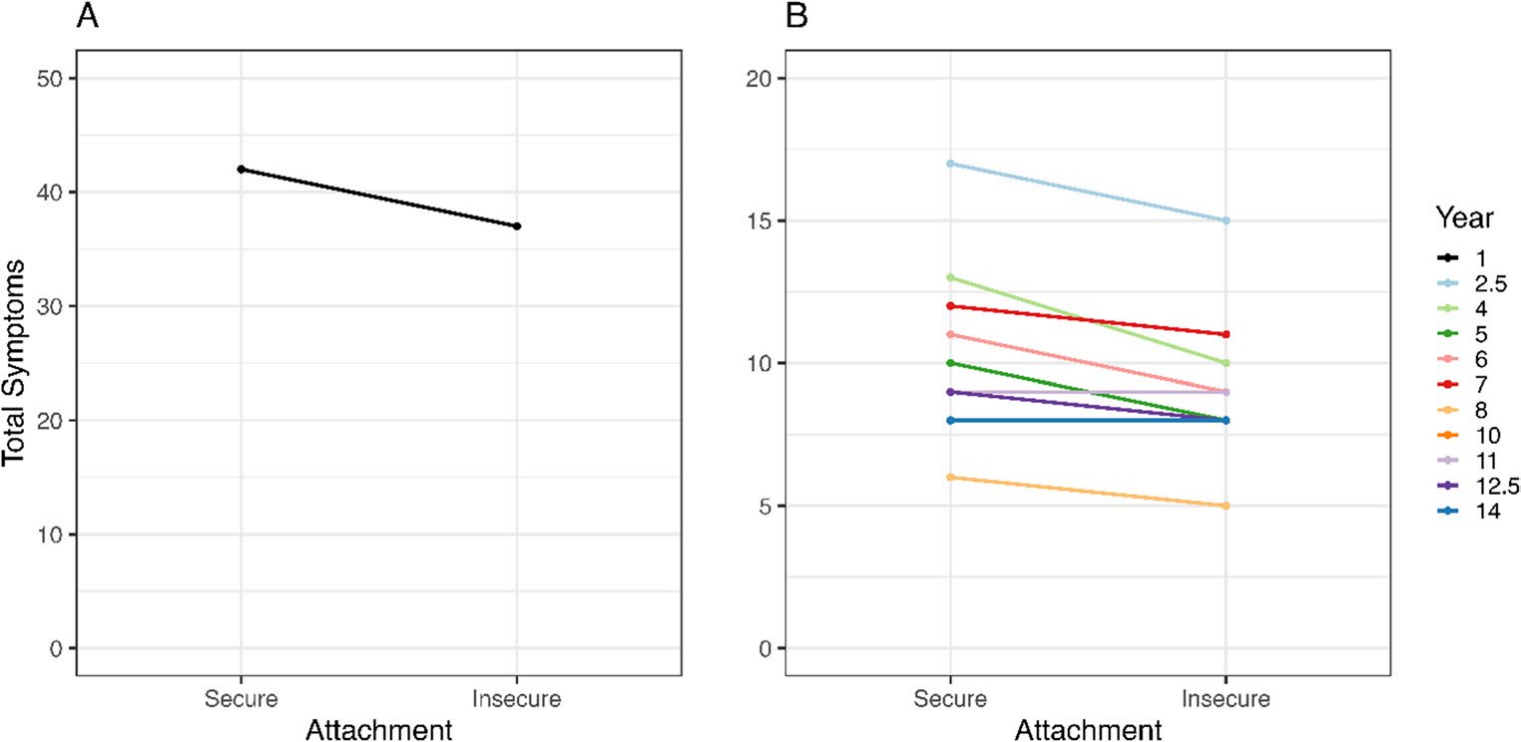
Median posterior predictions illustrate the effect size for the relation between attachment categories (x-axis) and total symptom counts (y-axis) for each year. The first year (**A**) is shown separately from the other years (**B**)

Using the continuous score for early life attachment security, we could not reject the null hypothesis for any of the symptoms. When we used the attachment categorization into *secure* and *insecure*, we found that mothers of insecurely attached children reported fewer symptoms for respiratory ($\:\beta\:$=−0.184, 95% CI=[−1.016; −0.049], *p* = 0.990) and general ($\:\beta\:$=−0.189, 95% CI=[−0.469; −0.062], *p* = 0.992) symptoms. Figure 4 shows that mothers of insecurely attached children are expected to report 5 (Fig. 4A) and between 0 and 3 (Fig. 4B) fewer total symptoms compared to mothers of children with secure attachment in the first and subsequent years, respectively. The exploration of the four attachment categories showed that securely attached children are estimated to have higher total symptoms than avoidant or resistant types. In contrast, infants with disorganized attachment are estimated to have similar total symptoms as the securely attached children.

#### Maternal sensitivity and infant-mother attachment

**Fig. 5:**
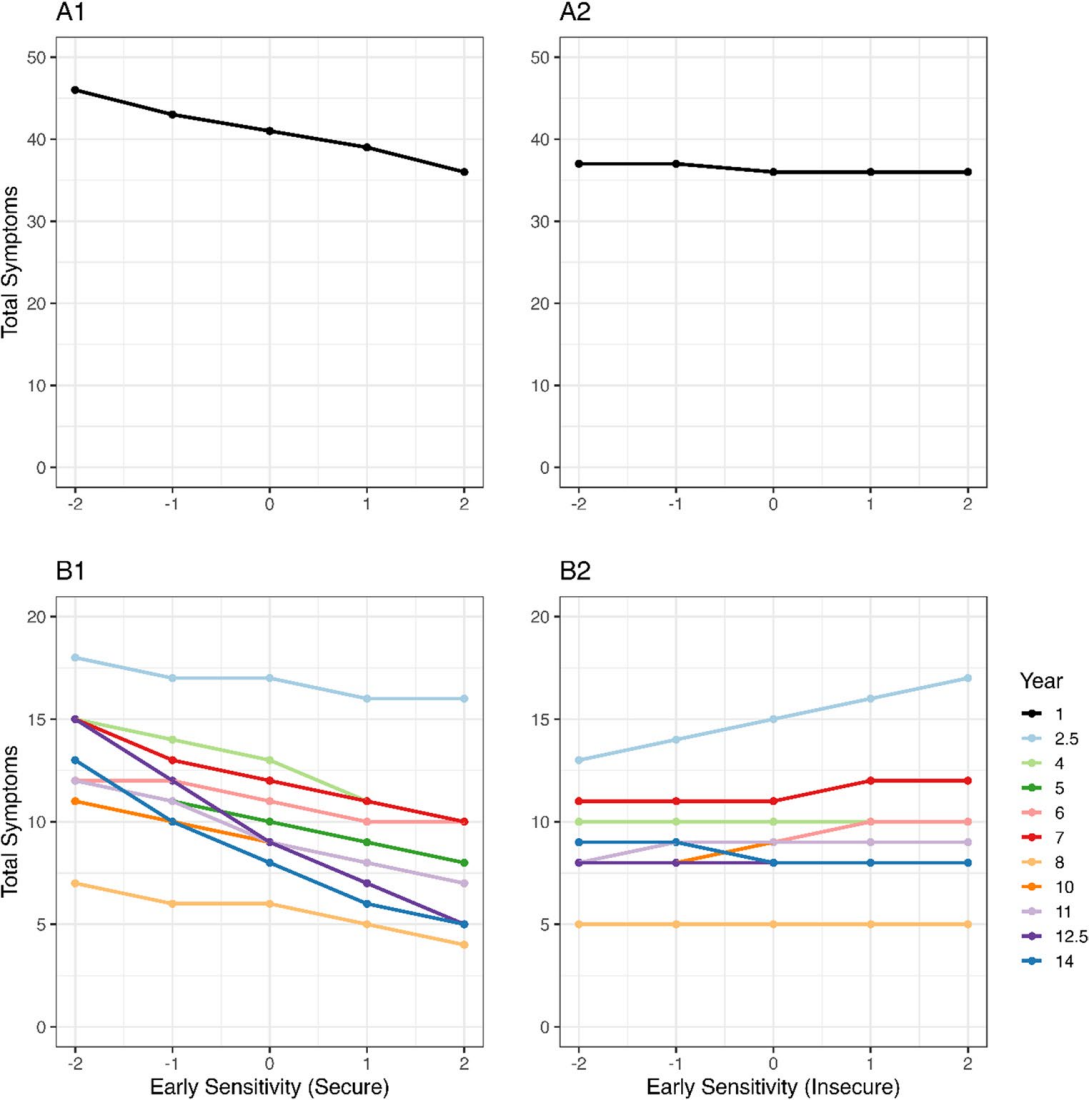
Posterior predictions illustrating the effect size for the relation between early maternal sensitivity in the first year (x-axis) and symptom counts (y-axis) per attachment category (secure vs. insecure). The first year (**A**) is shown separately from the other years (**B**)

In the final models, we included maternal sensitivity and attachment security (secure vs. insecure). The main effects remained in line with the results reported above. However, we found that early maternal sensitivity was only negatively associated with health symptoms among the children of mothers of securely attached children ($\:\beta\:$=0.132, 95% CI=[0.009; 0.470], *p* = 0.970). Accordingly, as maternal sensitivity increased from − 2SD to +2SD, the effect size of the relationship between maternal sensitivity and health symptoms became larger within the group of securely attached children with an expected decrease of 10 (Fig. 5A1) and between 2 and 10 (Fig. 5B1) symptoms, compared to a decrease of 1 (Fig. 5A2) and even an increase to up to 4 symptoms (Fig. 5B2) in insecure children.

**Fig. 6:**
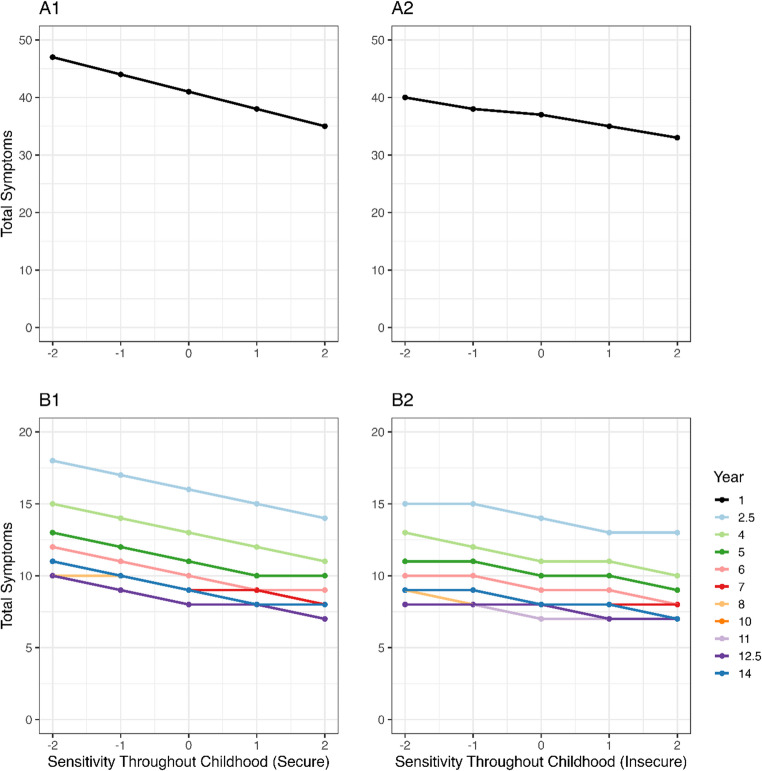
Posterior predictions illustrating the effect size for the relation between maternal sensitivity throughout childhood measured at several times points (x-axis) and symptom counts (y-axis) per attachment category (secure vs. insecure). The first year (**A**) is shown separately from the other years (**B**)

Interestingly, this interaction effect was not present, or was at least much smaller, when looking at maternal sensitivity throughout childhood. Here, maternal sensitivity remained associated with reported health symptoms across groups of attachment security. The relation still appeared stronger for the securely attached children, with an expected decrease of 10 (Fig. 6A1) and between 3 and 5 (Fig. 6B1), compared to 7 (Fig. 6A2) and between 2 and 3 (Fig. 6B2) total symptoms in the first and subsequent years, respectively.

## Discussion

In this study, we investigated the relationship between caregiving quality, defined as maternal sensitivity during infancy and throughout childhood, and attachment security in infancy, as well as children’s physical health over a span of 14 years, using Bayesian generalized linear mixed models. These analyses constitute a partial replication and significant extension of previous findings in the same cohort (Stern et al., 2020) by employing a Bayesian rather than a frequentist approach and expanding the timeline from 1 to 14 years of life. To this end, we utilized a total of five repeated measures of sensitivity along with an additional 11 measures of physical health. Results indicated that higher maternal sensitivity during the first year of life (5 weeks and 12 months of infant age), but not attachment security at 12 months, was associated with significantly fewer health complaints (i.e., respiratory issues) throughout the 14 years. Similarly, the main analysis revealed that maternal sensitivity throughout childhood (from 5 weeks to 14 years) was even more strongly correlated with fewer health complaints (i.e., total, respiratory, and digestive). Furthermore, our exploratory analyses demonstrated that higher early maternal sensitivity (during the first year of life) was linked to fewer symptoms over 14 years in the securely attached group only. In contrast, higher maternal sensitivity throughout childhood was associated with better health outcomes, regardless of attachment security classification. These effects persisted after adjusting for key covariates, including breastfeeding duration, center-based childcare attendance, number of siblings, infant sex, and socioeconomic status. Additional exploratory analyses on different attachment classifications revealed that mothers of securely attached children reported more symptoms than those of avoidant or resistant attachment types. Below, we elaborate on and interpret our findings.

### Maternal sensitivity and physical health

Although we did not directly measure stress regulation in this study, our results indicating an association between maternal sensitivity during infancy and childhood and reduced health complaints over 14 years are in line with the notion that mothers with higher levels of sensitivity may enhance their children’s stress regulation capabilities, hence reducing their susceptibility to illness (Loman & Gunnar, 2010; Pietromonaco & Beck, 2019). Research has shown that ongoing stress triggers inflammatory responses and weakens the immune system’s ability to fight pathogens (Glaser & Kiecolt-Glaser, 2005). Therefore, less sensitive caregiving might compromise children’s immune function, increasing their vulnerability to illness (Bilbo & Schwarz, 2009). Alternatively, it is conceivable that mothers who exhibit greater attentiveness and cooperation are more attuned to their children’s signals of distress or discomfort (e.g., Vacaru et al., 2022), and by responding promptly, prevent potential mild health issues from becoming serious symptoms. Maternal sensitivity, whether during the early years or over the 14 years, was consistently associated with fewer total complaints, particularly respiratory symptoms. Our findings build upon previous research within the same cohort conducted by Stern and colleagues (2020). A notable difference is that while the prior study found that sensitive care during infancy was associated with fewer skin complaints in the first year of life, in the current investigation, we could not reject the null hypothesis for skin symptoms. Instead, we found a link between maternal sensitivity throughout childhood and fewer digestive complaints. These variations in outcomes can be attributed to methodological distinctions between this study and the earlier one: (1) a more extended period of health assessments (14 years vs. one year), (2) a composite score for early maternal sensitivity encompassing assessments at 5 weeks and 12 months instead of solely at 5 weeks, and (3) the use of generalized linear mixed models (Poisson distribution) as opposed to linear mixed models (Gaussian distribution) in the previous study. In contrast to the findings regarding skin symptoms, the consistent association between maternal sensitivity and respiratory symptoms underscores the robustness of this observed effect. Further studies in this direction are needed to shed light on the specific mechanisms through which maternal sensitivity might specifically impact respiratory symptoms.

Interestingly, our findings align with emerging psychoneuroimmunological theories about how early caregiving shapes physical health. Maternal sensitivity likely influences immune development through various pathways involving allostatic load—the cumulative physiological burden arising from repeated stress responses (McEwen, 1998). Sensitive caregiving acts as a buffer against stress, preventing excessive allostatic load that can dysregulate immune function through prolonged exposure to stress hormones (McEwen & Stellar, 1993). Children who experience responsive caregiving typically show healthier cortisol reactivity patterns—more moderate responses to stressors and a swift return to baseline—which protect against immune suppression associated with chronic HPA axis dysregulation (Kuhlman et al., 2017, 2018). A meta-analysis by Groh and Narayan (2019) confirms that attachment insecurity is related to distinct patterns of physiological reactivity to interpersonal stress, supporting our findings of differential health outcomes between attachment patterns. Specifically, insecure-avoidant children often display blunted cortisol reactivity despite physiological arousal, which could lead to immune suppression where inflammation occurs without a corresponding behavioral manifestation of symptoms (Hostinar et al., 2014; Gunnar & Quevedo, 2008). Conversely, secure attachment may promote appropriate immune responses where symptoms are both expressed and addressed, possibly explaining our finding of increased reported symptoms despite theoretically better immune functioning (Berens et al., 2017).

### Attachment and physical health

Our findings on attachment are unexpected and contradict the results on maternal sensitivity. Data showed that mothers of children classified as insecure at 12 months of age reported fewer health complaints over a 14-year span. This outcome contrasts with the neuroendocrine perspective, which led us to anticipate that securely attached children would exhibit fewer health complaints, reflecting the effects of higher maternal sensitivity. In interpreting these results, it is essential to note that our analysis unveiled a lack of significant association between maternal sensitivity and attachment security in this sample. This finding is somewhat surprising, as it is widely accepted that maternal sensitivity plays a fundamental role in children’s attachment security, as evidenced by previous meta-analyses (see Atkinson et al., 2000; De Wolff & Van Ijzendoorn, 1997). The absence of a significant association between maternal sensitivity and attachment security in our sample warrants consideration. While this finding diverges from meta-analytic evidence establishing a moderate link between these constructs (De Wolff & Van Ijzendoorn, 1997; Verhage et al., 2016), our assessment methods were robust, including five repeated sensitivity measurements and a gold-standard attachment assessment. This unexpected finding likely reflects a combination of methodological and developmental factors. Sensitivity appears less stable across development than previously assumed (e.g., Bigelow et al., 2010), as shown by weak correlations between our repeated measurements. Cultural factors in this Dutch sample may also play a role, as parenting practices and their interpretations vary across contexts (Mesman et al., 2016), potentially altering the sensitivity-attachment relationship compared to the predominantly North American samples in which this association was established. This finding underscores that within our study sample, maternal sensitivity and child attachment may capture somewhat independent dimensions of caregiving quality. There are at least two possible explanations for the finding that mothers of insecurely attached children reported fewer health complaints. First, it is conceivable that mothers of insecurely attached children may have difficulty recognizing or interpreting symptoms and distress signals, or may be less observant and attentive to their children’s health symptoms, thereby underreporting health complaints. Alternatively, insecurely attached children may not effectively signal their needs or distress regarding symptoms to their mothers, compared to securely attached children. This speculation is supported by prior research that demonstrated that following a separation, infants displaying higher cortisol reactivity, particularly in the securely attached group, exhibited more self-soothing and fussing than their insecure counterparts (Beijers et al., 2017), suggesting that insecure infants did not signal their distress like secure infants. This explanation is also supported by the findings of Beijers and colleagues (Beijers, et al., 2011), who found that infant night waking patterns were associated with attachment classification, with insecure (avoidant) infants displaying significantly fewer parent-reported night wakings, possibly because upon waking at night, they signaled less to their parents, which, in turn, led to fewer reported night waking episodes. This interpretation aligns with attachment theory, wherein avoidant children develop strategies to minimize expressions of distress to avoid maternal rejection, while resistant children may exhibit inconsistent signaling patterns (Cassidy & Kobak, 2015). The differentiated health reporting patterns we observed across attachment classifications—with lower reported symptoms in avoidant and resistant but not disorganized children—support this interpretation, as disorganized attachment lacks the organized strategy for distress management seen in other insecure patterns. These findings highlight a methodological challenge in health research: parent-reported outcomes may reflect not just objective health status but also the complex interplay between child signaling behaviors and parental perception, which are themselves shaped by attachment dynamics (DeOliveira et al., 2005). Therefore, the current findings regarding attachment classifications, particularly the reduced health complaints in avoidant and resistant children compared to secure and disorganized classifications, highlight an interesting avenue for further investigation. Given the limited and uneven representation of children within each category, this discovery underscores the need for replication in larger studies to clarify these intriguing patterns.

While our study examined a low-risk sample of children through early adolescence, other important research has investigated relationships between early caregiving quality and health outcomes in high-risk adult populations. These studies have primarily focused on adults who experienced significant early adversities, including poverty, maltreatment, family dysfunction, or trauma exposure. For example, research with the Dunedin Multidisciplinary Health and Development Study found that adverse childhood experiences were associated with elevated inflammatory markers and poorer adult health outcomes (Danese et al., 2007). Similarly, the ACE Study demonstrated dose-response relationships between childhood adversities and adult health problems (Felitti et al., 1998). Our findings complement this adult high-risk literature by suggesting that even within low-risk samples with normative ranges of caregiving, variations in sensitivity may influence physical health development, potentially through similar but less pronounced mechanisms as those observed in high-risk contexts. Whether differences between our findings and adult high-risk samples are purely methodological or indicative of distinct underlying mechanisms remains a subject for future research, necessitating the alignment of methodologies and replication of our findings in diverse developmental samples. Subsequent investigations should also explore potential cultural variations in stress regulation and inflammatory processes, which could influence health outcomes. Prior work found considerable differences between Dutch and US samples in psychobiological stress regulation (i.e., infants, Sung et al., 2015); children and adolescents (Vacaru et al., 2023); and adult samples (Gartstein et al., 2020). Equally relevant is the difference in physical health assessment methods across the studies discussed above. In our study, we used maternal reports due to the intensive number of assessments (i.e., a total of 23 from birth to 14 years). In addition, we aimed to capture mild complaints that typically do not necessitate medical intervention, such as a sore throat or fever. In the local healthcare, it is uncommon for parents to report such mild symptoms to the general practitioner. In this context, parental reports might offer more complete data on general health symptoms in children. Future work, especially throughout adulthood, when such mild symptoms may progress into more serious conditions, should also consider adding medical records to self-reports on symptoms to obtain a more complete assessment of health.

### The interactive effect of maternal sensitivity and attachment security on physical health

Our study revealed that high maternal sensitivity in the first year was related to fewer health symptoms in securely attached infants, a group forming about 65% of our sample. This correlation persisted up to 14 years, unlike in insecurely attached infants, where maternal sensitivity in the first year was unrelated to health. It could be the case that the lack of an early secure attachment to the mother may override any potential additional effects of sensitive care on health symptoms. The difference between secure and insecure infants with respect to the role of caregiver’s sensitivity for physical health might be due to their early expectations of caregiver responsiveness, with insecure infants expecting less prompt care (Jia et al., 2023). Accordingly, insecure infants might exhibit altered stress and immune responses, including suppressed inflammatory reactions, similar to findings in adults (Picardi et al., 2013). However, more developmental psychobiological research is necessary to confirm these theories.

In contrast, greater maternal sensitivity throughout childhood, from 5 weeks to 14 years, was linked to fewer health complaints in both securely and insecurely attached groups. This finding may reflect that maternal sensitivity throughout childhood, as an index of caregiving history throughout different developmental phases, each with different demands on parents and the necessity for adaptation to children’s continuously changing needs, impacts children’s health irrespective of the earliest attachment to their mother. The contrary could also hold true: children who are healthier may be easier to attend to in a sensitive manner. Parents of children who are often or chronically sick may undergo more stress themselves, lowering their capacity to be sensitive (Teicher et al., 2020). Moreover, as they grow, children form attachment relations to other figures, such as teachers (Bergin & Bergin, 2009) or peers (Gorrese & Ruggieri, 2013), which may also significantly contribute to children’s stress regulation and illness vulnerability. Importantly, these findings warrant caution, given the exploratory nature of this analysis and the lower statistical power underlying the interaction effect.

### Strengths and limitations

This study builds upon a substantial sample, longitudinally followed from birth until 14 years of age, with a myriad of lab-based, home-based, and online parent-report and gold-standard observational measures. Our observational sensitivity measure is particularly robust, encompassing data collected at five different time points, from the infant’s fifth week of life to the age of 14. Notably, sensitivity measures did not offer evidence for greater stability, revealing small, significant correlations only between 5 weeks and 12 months and 10 and 14 years, in line with other findings (Yang et al., 2024; van der Voort et al., 2014). Not many studies used similar measures of sensitivity across a large time span to provide more contextualization on the stability of sensitivity. One study provided longitudinal evidence for small to moderate associations for some but not all waves with four measures of sensitive parenting throughout the first 15 years of life (Zvara et al., 2018). Similarly, physical health assessments involved a meticulous approach, entailing 12 monthly-based evaluations within the first year of life, followed by 10 additional assessments occurring roughly at yearly intervals throughout the child’s development from 0 to 14 years. It is worth noting that the higher symptom count observed in the first year, in comparison to the subsequent years where symptom counts remain relatively stable, can be attributed to the measurement methodology. Our statistical models accounted for these differences, ensuring that these variations did not impact our maternal sensitivity and attachment estimates. Furthermore, we acknowledge that our final sample size of 150 participants is modest for complex longitudinal analyses with multiple covariates. However, several factors strengthen our analytical approach. Our Bayesian statistical framework is well-suited for handling smaller samples with complex models, as it does not rely on large-sample asymptotics (Gelman et al., 2013). Furthermore, our statistical power is enhanced by the repeated-measures design with 23 health assessments per participant, yielding over 3,000 observations for analysis. The consistency of findings across different outcomes and models suggests reliable effects despite the moderate sample size. Nevertheless, replication with larger samples would be valuable to confirm the generalizability of these results. A limitation of our study is that the mother was the sole reporter for child health, which may introduce bias, especially in parents whose infants are classified as insecure. This may have influenced our findings that mothers of insecure infants reported fewer symptoms. Future work should complement maternal reports of child health with medical reports, child self-reports, or another caregiver’s reports to gather a more comprehensive depiction of the child’s health status. In addition, we propose that assessing attachment to the second primary caregiver may provide additional insights, as attachment relationship quality may differ between the two parents, and one may buffer the effects of the other or enhance an effect for better or for worse (Dagan & Sagi-Schwartz, 2018). Finally, our findings indicate that enhancing maternal sensitivity through evidence-based interventions, such as video-feedback techniques (Juffer et al., 2017) or attachment-based parenting programs (Bernard et al., 2012), next to improving child socio-emotional outcomes, could also potentially improve children’s physical health outcomes, particularly regarding respiratory and digestive symptoms. Healthcare systems could incorporate brief sensitivity assessments during well-child visits, enabling early identification and support for parent-child dyads who might benefit from targeted interventions that could ultimately reduce healthcare utilization and improve children’s long-term health trajectories (Cassidy et al., 2017; Ehrlich et al., 2016).

## Conclusion

This study sheds light on the potential importance of typical variations in caregiving quality, including maternal sensitivity and attachment, to child health. Higher early maternal sensitivity in the first year of an infant’s life and sensitivity throughout 14 years were related to fewer total health complaints and notably fewer respiratory and digestive symptoms. When early maternal sensitivity was higher, securely attached children showed fewer symptoms, while insecurely attached children showed fewer health complaints, irrespective of maternal sensitivity throughout childhood. These findings indicate that parental behavior towards the child may impact a child’s physical health. The notion that parental behavior, even within typical or low-risk contexts, may play a vital role in safeguarding a child’s health and well-being may inspire future research into underlying mechanisms and a holistic approach to pediatric healthcare.

## Supplementary Information

Below is the link to the electronic supplementary material.

Supplementary Material 1

## Data Availability

The data necessary to reproduce the analyses presented here are not publicly accessible due to the earliest signed informed consent that did not include information on public data sharing. Access to the data can be obtained upon request from the data access manager, Irene van Kroonenburg. The analytic code necessary to reproduce the analyses presented in this paper is publicly accessible (https://doi.org/10.5281/zenodo.10401249). The materials necessary to replicate the findings presented here are not publicly available but can be requested from the data access manager, Irene van Kroonenburg . The analyses presented here were preregistered (https://aspredicted.org/tt55z.pdf) .
