# Supplementary material for: A healthy start: examining the contribution of caregiving quality to child physical health from birth to 14 years
Source: Curr Psychol. 2025 Sep 6;44(21):17021–39. doi: 10.1007/s12144-025-08350-5 (PMC12586213; doi:10.1007/s12144-025-08350-5)
Supplement: Supplementary file 1 — Supplementary Material 1 [file 12144_2025_8350_MOESM1_ESM.docx]

**Supplementary material**


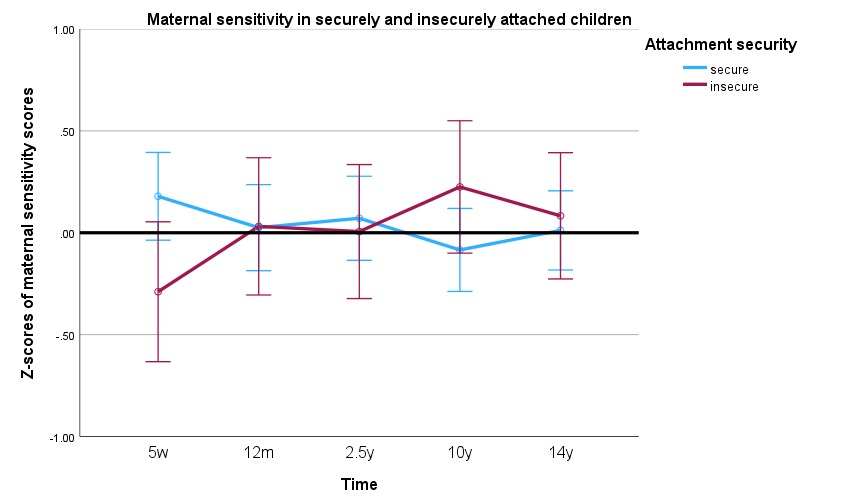


**Supplementary Figure 1.** Maternal sensitivity in secure and insecure children across time. The error bars represent the 95% confidence interval.

| **Supplementary Table 1.** Raw scores of maternal sensitivity across ages and per attachment category. | | | | |
| --- | --- | --- | --- | --- |
|  | **Avoidant** | **Secure** | **Resistant** | **Disorganized** |
|  | *M(SD)* | *M(SD)* | *M(SD)* | *M(SD)* |
| 5 weeks | 5.22 (2.61) | 5.58 (2.04) | 5.30 (1.93) | 5.17 (2.13) |
| 12 months | 4.66 (1.11) | 4.56 (.98) | 4.87 (1.19) | 4.30 (.98) |
| 2.5 years | 5.53 (.56) | 5.51 (.67) | 5.42 (.68) | 5.39 (.70) |
| 10 years | 5.52 (.56) | 5.52 (.81) | 5.76 (.69) | 5.58 (.59) |
| 14 years | 4.36 (1.91) | 5.13 (1.12) | 5.20 (1.00) | 5.22 (1.42) |
| *Notes.* All sensitivity scores range from 1-7 except for the 5-week assessment, which ranges from 1-9. | | | | |

| **Supplementary Table 2*.*** Composite health scores per category and the total health score per child age. | | | |
| --- | --- | --- | --- |
|  | **Mean** | **SD** | **Range** |
| 1 year^a^ (*n=*188)  *Respiratory*  *General*  *Digestive*  *Skin*  *Total Health* | 26.93  6.78  5.07  5.88  44.66 | 8.75  3.73  3.94  4.17  13.81 | 0.0 – 53.5  0.0 – 18.0  0.0 – 19.0  0.0 – 23.5  6.00 – 88.00 |
| 2.5 years (*n=*178)  *Respiratory*  *General*  *Digestive*  *Skin*  *Total Health* | 6.84  4.08  5.33  1.76  17.81 | 5.76  3.31  5.62  3.72  11.13 | 0.0 – 30.00  0.0 – 17.0  0.0 – 36.0  0.0 – 13.0  0.0 – 62.50 |
| 4 years (*n=*179)  *Respiratory*  *General*  *Digestive*  *Skin*  *Total Health* | 5.56  2.58  3.30  1.51  12.96 | 5.27  2.51  4.22  3.45  9.91 | 0.0 – 30.0  0.0 – 15.0  0.0 – 19.0  0.0 – 12.0  0.0 – 48.50 |
| 5 years (*n=*176)  *Respiratory*  *General*  *Digestive*  *Skin*  *Total Health* | 3.61  2.30  3.01  1.16  10.05 | 4.52  2.66  4.42  3.17  9.75 | 0.0 – 27.0  0.0 – 20.0  0.0 – 24.0  0.0 – 12.0  0.0 – 56.00 |
| 6 years (*n=*160)  *Respiratory*  *General*  *Digestive*  *Skin*  *Total Health* | 4.47  2.22  3.32  1.36  11.45 | 3.88  2.14  3.49  3.07  8.09 | 0.0 – 20.0  0.0 – 12.0  0.0 – 19.0  0.0 – 13.0  0.0 – 43.00 |
| 7 years (*n=*171)  *Respiratory*  *General*  *Digestive*  *Skin*  *Total Health* | 3.66  2.70  5.14  1.83  13.32 | 4.53  2.47  5.17  3.97  10.11 | 0.0 – 34.00  0.0 – 12.00  0.0 – 28.00  0.0 – 20.00  0.0 – 55.00 |
| 8 years (*n=*172)  *Respiratory*  *General*  *Digestive*  *Skin*  *Total Health* | 2.74  1.38  0.76  1.28  6.15 | 2.95  1.52  1.68  2.97  5.40 | 0.0 – 16.00  0.0 – 9.00  00 – 20.00  0.0 – 12.00  0.0 – 25.00 |
| 10 years (*n=*149)  *Respiratory*  *General*  *Digestive*  *Skin*  *Total Health* | 2.90  1.77  4.24  1.11  10.01 | 2.61  2.17  4.92  3.30  7.70 | 0.0 – 18.00  0.0 – 15.00  0.0 – 27.00  0.0 – 24.00  0.0 – 37.00 |
| 11 years (*n=*136)  *Respiratory*  *General*  *Digestive*  *Skin*  *Total Health* | 2.82  1.85  3.67  1.76  10.10 | 3.14  1.88  4.49  4.62  8.90 | 0.0 – 24.00  0.0 – 11.00  0.0 – 20.00  0.0 – 25.00  0.0 – 44.00 |
| 12.5 years (*n=*150)  *Respiratory*  *General*  *Digestive*  *Skin*  *Total Health* | 4.59  1.26  3.16  1.04  10.04 | 6.80  1.87  5.69  3.59  11.36 | 0.0 – 48.00  0.0 – 13.00  0.0 – 39.50  0.0 – 24.00  0.0 – 64.50 |
| 14 years (*n=*150)  *Respiratory*  *General*  *Digestive*  *Skin*  *Total Health* | 3.16  0.76  3.16  2.03  9.11 | 5.28  1.35  6.25  6.11  11.03 | 0.0 – 49.00  0.0 – 12.00  0.0 – 38.00  0.0 – 25.00  0.0 – 53.50 |
| *Notes.* The mean health scores represent the sum of the symptoms/illnesses in the year preceding the assessment. N=number of participants; SD=standard deviation. ^a^ Numbers in the first year are not comparable to those of the remaining years because of the different data collection frequency, while all subsequent years are comparable (see Methods). | | | |

**Supplementary Table 3.** Summary of the posterior distribution of all beta coefficients as estimated by the Bayesian Generalized Linear Models. Results are grouped by the different independent variables.

| **DV** | **β** | **95% CI** | **p** |
| --- | --- | --- | --- |
| **Maternal Sensitivity First Year** | | | |
| Respiratory | -0.068 | [-0.203; -0.009] | 0.981 |
| Skin | -0.061 | [-0.454; 0.079] | 0.785 |
| Digestive | -0.036 | [-0.348; 0.073] | 0.701 |
| General | -0.008 | [-0.154; 0.057] | 0.608 |
| Total | -0.043 | [-0.769; 0.008] | 0.949 |
| **Maternal Sensitivity Throughout Childhood** | | | |
| Respiratory | -0.110 | [-0.348; -0.040] | 0.994 |
| Skin | -0.005 | [-0.560; 0.179] | 0.481 |
| Digestive | -0.142 | [-0.475; -0.015] | 0.955 |
| General | -0.025 | [-0.333; 0.063] | 0.660 |
| Total | -0.095 | [-0.440; -0.022] | 0.988 |
| **Attachment Security** | | | |
| Respiratory | 0.021 | [-0.030; 0.043] | 0.946 |
| Skin | -0.010 | [-0.147; 0.049] | 0.369 |
| Digestive | 0.005 | [-0.081; 0.041] | 0.589 |
| General | 0.016 | [-0.055; 0.042] | 0.889 |
| Total | 0.013 | [-0.037; 0.034] | 0.880 |
| **Attachment Security Dummy** | | | |
| Respiratory | -0.184 | [-1.016; -0.049] | 0.990 |
| Skin | 0.014 | [-0.695; 0.330] | 0.480 |
| Digestive | -0.063 | [-0.668; 0.145] | 0.701 |
| General | -0.189 | [-0.469; -0.062] | 0.992 |
| Total | -0.106 | [-0.421; 0.019] | 0.938 |

*Notes.* DV = Dependent Variable, 95% CI = 95% Credible Interval, p = Probability that β is smaller or larger than 0, depending on the direction of the hypothesis.
